# The Application Effect of Traditional Chinese Medicine Nursing on General Anesthesia Combined with Epidural Anesthesia and Electric Resection for the Treatment of Bladder Cancer and Its Influence on Tumor Markers

**DOI:** 10.1155/2022/7178711

**Published:** 2022-01-15

**Authors:** Yan Wu, Zhenna Zhang, Yangfan Liu, Guangwen Shi, Xuehai Ding

**Affiliations:** ^1^Department of Clinical Laboratory, Yantai Yuhuangding Hospital, Yantai 264000, China; ^2^Outpatient Department, Affiliated Qingdao Central Hospital, Qingdao University, Qingdao 266042, China; ^3^Health Management Center, Zhangqiu District People's Hospital, Jinan 250200, China; ^4^Department of Anesthesiology, Qingdao Hospital of Traditional Chinese Medicine, Qingdao Hiser Hospital, Qingdao 266033, China

## Abstract

**Objective:**

To explore the effects of traditional Chinese medicine nursing on general anesthesia combined with epidural anesthesia and electric resection to treat bladder cancer and its influence on tumor markers.

**Methods:**

A total of 160 patients with non-muscle-invasive bladder cancer who underwent general anesthesia combined with epidural anesthesia and resection were included in this study. The patients were divided into control group (*n* = 80) and study group (*n* = 80) according to the random number table method. The control group received hydroxycamptothecin bladder perfusion therapy, and the study group received traditional Chinese medicine nursing combined with hydroxycamptothecin bladder perfusion therapy. The clinical efficacy, three-year cumulative survival rate, and postoperative recurrence rate of the two groups of patients were detected. The levels of tumor markers including vascular endothelial growth factor (VECF) and bladder tumor antigen (BTA) before and after treatment were also tested. The immune function, inflammatory factor levels, and quality of life of the two groups before and after treatment were evaluated.

**Results:**

The total effective rate of the study group (83.75%) was significantly higher than that of the control group (58.75%). After treatment, the serum VEGF and BTA levels, inflammatory factors interleukin-6 (IL-6), C-reactive protein (CRP), and tumor necrosis factor-*α* (TNF-*α*) levels of the two groups of patients decreased, and the decrease in the study group was more significant than that in the control group (*P* < 0.05). After treatment, the levels of CD3+, CD4+, and CD4+/CD8+ in the two groups increased (*P* < 0.05), and the increase in the study group was more significant than that in the control group (*P* < 0.05). After treatment, the CD8+ levels of the two groups of patients decreased (*P* < 0.05), and the decrease in the study group was more significant than that in the control group (*P* < 0.05). After treatment, the quality-of-life scores in both groups increased (*P* < 0.05), and the increase in the study group was even more significant (*P* < 0.05).

**Conclusion:**

Traditional Chinese medicine nursing has significant clinical effects on the treatment of bladder cancer with general anesthesia combined with epidural anesthesia and electric resection. It can more effectively prevent the risk of recurrence of bladder cancer after surgery, significantly improve the quality of life, improve immune system function, regulate the levels of VECF and BTA, effectively reduce the level of serum inflammatory factors, inhibit tumor progression, and reduce tumor viability.

## 1. Introduction

Bladder cancer is one of the common clinical urinary system malignancies; its cause is long-term exposure to chronic infection, environment, foreign body stimulation, and other factors, leading to inactivation of tumor suppressor genes and activation of oncogenes, which in turn induce bladder cancer [1]. The early clinical manifestations of bladder cancer are symptoms such as frequent urination, urgency, and hematuria, and they are nonspecific. A few patients only have abdominal pain, and the rate of misdiagnosis and missed diagnosis is high [2]. Clinically, it can be classified into non-muscle-invasive bladder cancer (NMIBC), muscle-invasive bladder cancer, and metastatic bladder cancer [3]. NMIBC is one of the most common pathological types, accounting for more than 70% of bladder cancer, with high incidence, and its incidence has been increasing year by year in recent years and has become a critical disease endangering human health [4]. Early detection and scientific and effective intervention measures can effectively control the progression and deterioration of the disease and improve patients' quality of life.

Radical surgical resection is the main treatment for NMIBC, and most patients can be cured by transurethral resection of bladder tumor (TURBT) [5]. The application of anesthesia is an indispensable part of the operation, and the effects of different anesthesia methods are different, which directly relates to whether the operation can be carried out smoothly. General anesthesia combined with epidural anesthesia is widely used in clinical practice. Under the premise of ensuring the effect of anesthesia, this method has a small amount of anesthetic, which helps to reduce the perioperative stress response [6]. According to reports, the recurrence rate of bladder cancer is high, and about 1/3 of them have progressed, which seriously affects the effect of surgical treatment [7, 8]. Western medicine routinely uses chemotherapy drugs to prevent the recurrence of bladder cancer after surgery; although it can reduce the risk of tumor recurrence to a certain extent, the overall effect is still unsatisfactory. Long-term use of drugs has obvious side effects, and many patients give up because they cannot bear the perfusion therapy [9]. Studies have shown that traditional Chinese medicine (TCM) has obvious synergistic and detoxification effects on patients with bladder cancer postoperative perfusion. It can promote the recovery of body functions by enhancing the body's righteousness, which can regulate the body's immune function as a whole, prolong survival time, and improve the quality of life of patients [10]. We aimed to study the effects of traditional Chinese medicine nursing on patients after surgery and the impact on tumor markers.

## 2. Materials and Methods

### 2.1. Clinical Information

From March 2014 to October 2016, 160 patients with non-muscle-invasive bladder cancer who underwent general anesthesia combined with epidural anesthesia and resection at Yantai Yuhuangding Hospital, Yantai, Shandong, China, were selected and randomly divided into control group and study group, with 80 cases in each group. Control group comprised 67 males and 13 females, with age 43–71 years, average (52.65 ± 7.32) years old; duration 0.4–2 years, average (0.93 ± 0.36) years. Study group comprised 64 males and 16 females, with age 42–69 years, average (51.24 ± 7.28) years; duration 0.5–2 years, average (0.98 ± 0.41) years. There was no statistically significant difference in general information between the two groups (*P* > 0.05).

#### 2.1.1. Inclusion Criteria

All were in line with the “Guideline Manual for the Diagnosis and Treatment of Urological Diseases in China” [11] Western Medicine Diagnostic Standards and “Integrated Traditional Chinese and Western Medicine Urology” [12] Damp Toxins and Qi Deficiency Syndrome Differentiation Diagnosis Standards in Traditional Chinese Medicine; all were operated on pathologically confirmed, aware of this study, and signed informed consent; patients were without a history of radiotherapy, chemotherapy, and immunotherapy; expected survival time is more than 6 months.

#### 2.1.2. Exclusion Criteria

Patients with autoimmune diseases, blood system diseases, systemic infectious diseases, or tumors in other parts; patients with stones or urinary tract infections; patients with severe mental diseases such as bipolar disorder, poor compliance, or other factors which makes it difficult to complete this research were excluded. This study was approved by the Ethics Committee of the Yantai Yuhuangding Hospital, Yantai, Shandong, China.

### 2.2. Treatment Methods

The control group was given bladder infusion of hydroxycamptothecin (Hubei Li Pharmaceutical Co., Ltd., National Medicine Standard H20033896, 2 mL: 5 mg). 40 mg was added to 40 mL of 0.9% medical sodium chloride injection and then injected into the bladder through a catheter, respectively, maintaining the left, right, supine, and prone positions for 30 minutes each, once a week for 8 times, and then changing to once a month. The study group added traditional Chinese medicine nursing treatment on this basis. The prescriptions were as follows: *Hedyotis diffusa* 30 g, honeysuckle 30 g, comfrey 30 g, agrimony 25 g, *rehmannia* 20 g, Wangbuliuxing 15 g, plantago 15 g, angelica 10 g, 6 g of *Prunella vulgaris*, 6 g of licorice flakes, 6 g of *Panax notoginseng* powder, and 6 g of cork. Take 1 dose per day, add 300 mL of water, decoct to 100 mL, and take it warmly in the morning and evening. The treatment time for both groups was 12 months.

### 2.3. Observation Indicators

1. Compare the clinical efficacy of the two groups [13]. The efficacy is divided into complete remission (CR), partial remission (PR), stable (SD), and disease progression (PD). Evaluation criteria are as follows: CR: all tumor lesions disappeared after treatment, or imaging examination showed that the lesion volume was reduced by more than 75%; PR: the lesion volume was reduced by 50% to 75%; SD: the lesion volume was reduced by 25% to 50%; PD: the size of the lesion decreased remained unchanged or expanded, and even new lesions were produced. Total effective rate is as follows: (CR + PR)/total number of cases×100%.
2. Compare the levels of tumor markers before and after treatment in the two groups. Enzyme-linked immunosorbent assay was used to detect the levels of vascular endothelial growth factor (VEGF) in patients. Radioimmunoassay was used to detect serum tumor markers including bladder tumor antigen (BTA) levels.
3. Compare the levels of inflammatory factors before and after treatment in the two groups. Enzyme-linked immunosorbent assay (ELISA) detects the levels of interleukin-6 (IL-6), C-reactive protein (CRP), and tumor necrosis factor-*α* (TNF-*α*). The test kit is produced by Nanjing Jiancheng Institute of Biological Engineering and is operated strictly according to the kit instructions.
4. Compare the quality of life between the two groups before and after treatment. EORTC QOL-C30 scale was used to score [14]. Refer to the “Guidebook for Diagnosis and Treatment of Urological Diseases in China” to calculate the scores of physical function, cognitive function, role function, social function, and emotional function. Each item is worth 100 points. The higher the score, the better the quality of life.
5. Compare the immune function of the two groups before and after treatment. Flow cytometry was used to detect the levels of CD3+, CD4+, CD8+, and CD4+/CD8+.
6. Compare the long-term efficacy and recurrence rate of the two groups. Call monthly for return visits within 3 years after surgery, and calculate the cumulative survival rate for three years. Cystoscopy should be reviewed every 3 months within 1 year after surgery, and cystoscopy should be reviewed every 6 months in the second and third years. If suspicious lesions are found during cystoscopy, random mucosal biopsy or pathological biopsy of suspicious tissues will be performed to determine whether it is a recurrence of bladder cancer and to make statistics on the recurrence rate.

### 2.4. Statistical Analysis

The data was analyzed and processed by SPSS 19.0. Measurement data were expressed as mean ± standard deviation (*x* ± *s*) and subjected to *t* test; count data were expressed as *n* (%) and subjected to *χ*^2^ test. *P* < 0.05 indicated that the difference was statistically significant.

## 3. Results

### 3.1. Comparison of Clinical Efficacy between the Two Groups of Patients after Treatment

The total effective rate of treatment in the study group was 83.75%, and the total effective rate in the control group was 58.75%. The total effective rate of the study group was significantly higher than that of the control group (*χ*^2^ = 14.706, *P*=0.02), as shown in Table 1.

### 3.2. Comparison of Tumor Marker Levels before and after Treatment between the Two Groups

**Figure 1:**
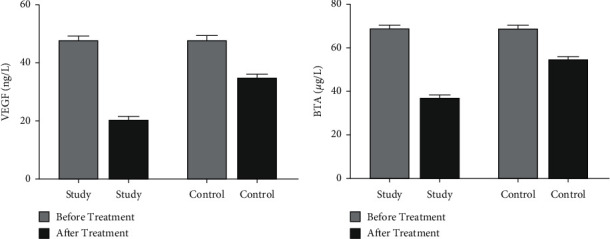
The levels of tumor markers in the two groups of patients before and after treatment. (a) The comparison of the VEGF levels of the two groups of patients before and after treatment. (b) The comparison of the BTA levels of the two groups of patients before and after the treatment.

Before treatment, there was no statistically significant difference in the levels of VEGF and BTA between the two groups of patients (*P* > 0.05). After treatment, the levels of both indexes of the two groups of patients decreased, and the decrease in the study group was more significant than that in the control group (*P* < 0.05), as shown in Figure 1.

### 3.3. Comparison of the Immune Function of the Two Groups of Patients before and after Treatment

**Figure 2:**
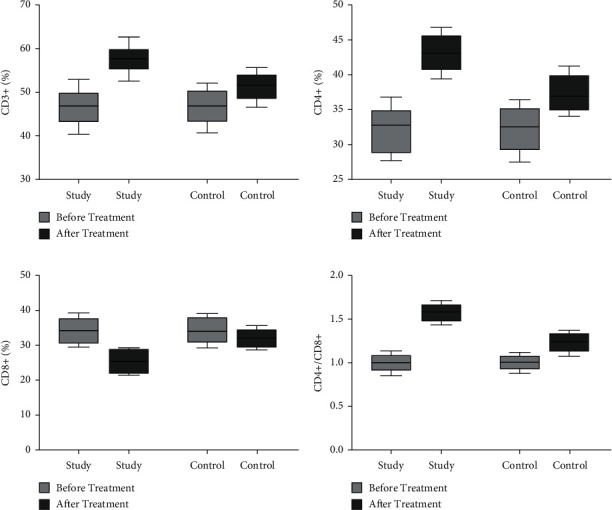
Comparison of the immune function of the two groups of patients before and after treatment. (a) The comparison of the CD3+ levels of the two groups of patients before and after treatment. (b) The comparison of the CD4+ levels of the two groups of patients before and after treatment. (c) The comparison of the CD8+ levels of the two groups of patients before and after treatment. (d) The comparison of CD4+/CD8+ levels before and after treatment in the two groups.

Before treatment, there was no significant difference in the levels of CD3+, CD4+, CD8+, and CD4+/CD8+ between the two groups. After treatment, the levels of CD3+, CD4+, CD8+, and CD4+/CD8+ in the study group were 57.61 ± 3.007, 43.19 ± 2.695, 25.40 ± 3.385, and 1.577 ± 1.104; the levels of CD3+, CD4+, CD8+, and CD4+/CD8+ in the control group were 51.28 ± 2.922, 37.34 ± 2.697, 32.15 ± 2.632, and 1.233 ± 0.111. The difference between the study group and the control group after treatment was statistically significant (*P* < 0.05, Figure 2).

### 3.4. Comparison of the Patients' Quality of Life in Two Groups before and after Treatment

Before treatment, there was no significant difference in the scores of physical function, role function, emotional function, cognitive function, and social function between the two groups of patients (*P* > 0.05). After treatment, the scores of various indicators in the two groups increased compared with those before treatment (*P* < 0.05), and the score of the study group increased significantly (*P* < 0.05, Table 2).

### 3.5. Comparison of the Levels of Inflammatory Factors before and after Treatment between the Two Groups

**Figure 3:**
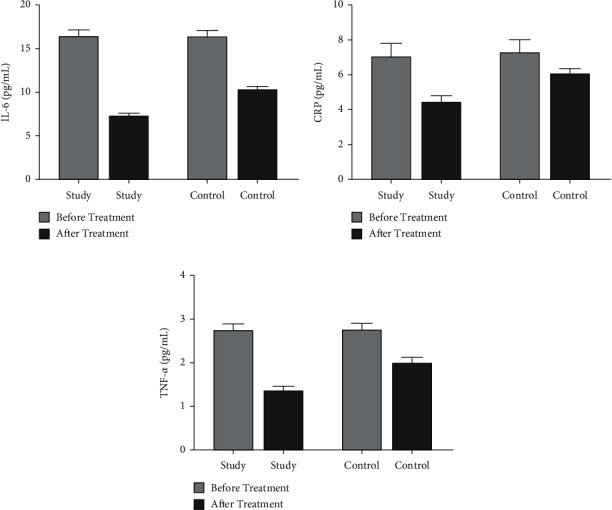
Comparison of the levels of inflammatory factors between the two groups of patients before and after treatment. (a) The comparison of IL-6 levels before and after treatment in the two groups of patients. (b) The comparison of CRP levels between the two groups of patients before and after treatment. (c) The comparison of TNF-*α* levels before and after treatment in the two groups.

Before treatment, there was no significant difference in the levels of IL-6, CRP, and TNF-*α* between the two groups of patients (*P* > 0.05). After treatment, the levels of three indicators of the two groups of patients decreased, and the decrease in the study group was more significant than that in the control group (*P* < 0.05, Figure 3).

### 3.6. Comparison of Long-Term Efficacy and Recurrence Rate between the Two Groups

**Figure 4:**
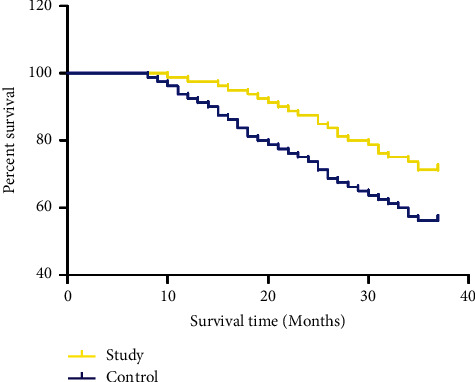
Comparison of the three-year cumulative survival rate of the two groups of patients.

After three years of follow-up, the three-year cumulative survival rate of the study group was 71.25%, and the recurrence rate was 32.50%; the three-year cumulative survival rate of the control group was 56.25%, and the recurrence rate was 61.25%. The difference between the two groups was statistically significant (*P* < 0.05, Figure 4, Table 3).

## 4. Discussion

Bladder cancer is a common primary malignant tumor of the urinary system in clinical practice, and its incidence has ranked first among urogenital tumors in China.

The main clinical manifestations of the patient are urinary discomfort, pain, hematuria with frequent urination, and other symptoms. As the disease becomes more serious, it could block the patient's urine flow and cause certain difficulties in urination [15]. The main reason for the occurrence of this disease is the dual effect of internal genetic factors and external environmental factors. Numerous studies have shown [4] that non-muscle-invasive bladder cancer accounts for more than 70% of initial bladder tumors and has the characteristics of strong infiltration, high malignancy, and easy recurrence. Surgery is currently the first choice for clinical treatment of this disease, and it effectively improves the patient's clinical symptoms and controls the progress of the disease. Because the bladder function can be kept normal, the postoperative recurrence rate is extremely high [16]. The tumor recurrence rate of 2 years after surgery is as high as 50%, and as much as 10% to 15% of patients with recurrent bladder cancer will experience varying degrees of deterioration. Some scholars pointed out [17] that early detection of bladder cancer and its recurrence is a key to successful cures and reducing mortality.

For the prevention of postoperative recurrence of patients with non-muscle-invasive bladder cancer, in Western medicine hydroxycamptothecin is mainly used as the primary chemotherapy drug for bladder perfusion therapy, which can reduce the recurrence rate caused by tumor cell dissemination after surgery, thereby effectively improving the therapeutic effects and improving the prognosis of patients [18]. The recurrence rate of bladder cancer with hydroxycamptothecin bladder perfusion is significantly lower than that of other chemotherapy drugs and its safety is higher [19]. It is currently one of the first-choice chemotherapy drugs for bladder perfusion in patients with bladder cancer. The toxic and side effects of hydroxycamptothecin are smaller than other chemotherapy drugs; however, because most patients are older at the time of diagnosis, their body is already in a declining period, and the body's immune capacity is significantly reduced after surgery. Severe infection due to severe reduction of white blood cells caused by the myelosuppressive reaction after perfusion leads to poor prognosis of patients.

In recent years, the application of TCM in the adjuvant treatment of malignant tumors has made great progress. TCM is used to prevent and treat bladder tumors at all stages of tumor development and has obvious advantages in improving clinical symptoms, improving quality of life, and reducing toxic side effects [20]. According to TCM concepts, malignant tumors are caused by a lack of righteousness, internal invasion of evil toxins, blocking of qi and blood, stagnation of qi, blood stasis, and accumulation of toxins [21]. According to the TCM, the main pathogenesis of bladder cancer is damp heat or damp water gathering in the lower body [22]. The prescription used in this study consists of *Oldenlandia diffusa*, honeysuckle vine, comfrey, agrimony, *Rehmannia glutinosa*, wangbuliuxing, plantain, angelica, prunella, licorice, *Panax notoginseng* powder, and cork. Among these, *Oldenlandia diffusa* clears heat and detoxifies, relieves pain, and dissipates agglomeration, diuresis, and dehumidification; *Prunella vulgaris* clears fire and eyesight; licorice clears heat and detoxifies; honeysuckle clears heat and detoxifies, activates blood, and clears collaterals; *Lithospermum* cools and activates blood, clears heat, and detoxifies; *Panax notoginseng* powder stops bleeding, disperses blood stasis, and relieves pain; *Rehmannia glutinosa* nourishes and cools blood; Cheqianzi benefits water and heat and clears heat; *Phellodendron chinense* clears heat and dampness, purges fire, and detoxifies; angelica replenishes blood, promotes blood circulation, nourishes the intestines, and makes bowel movement smooth; Wang buliuxing can promote blood circulation, detoxification and detumescence, diuresis, and gonorrhea; Agrimonia can stop bleeding and detoxify [23–27]. The combination of all medicines has the effects of clearing heat and detoxification, reducing swelling and blood stasis, stopping bleeding, and relieving pain.

VEGF is one of the essential cytokines that stimulate vascular endothelial cell proliferation and angiogenesis. Its increased expression level can promote endothelial cell differentiation and stimulate bladder tumor angiogenesis, which is closely related to the occurrence and development of bladder cancer and can be used to evaluate the malignancy of bladder tumors. It is an important reference index for prognosis [28]. Puntoni et al. [29] showed that VEGF is related to the clinicopathological characteristics of bladder cancer, which can reflect the development of bladder cancer patients to a certain extent, confirming that VEGF is closely related to the malignant development of bladder cancer patients, and it is expected to be used as a marker for prognostic evaluation. Zhu et al. [30] demonstrated that VEGF could promote tumor angiogenesis, regulate tumor cell proliferation, invasion, migration, and other malignant biological behaviors, and affect the prognosis and survival of patients with bladder cancer. Various studies have reported [31, 32] that, with the increase in the staging and grade of bladder tumors, the detection level of bladder tumor antigen (BTA) increases, the detection rate of multiple tumors is significantly higher than that of single tumors, and the initial tumor is significantly higher than that of single tumors, suggesting that BTA can be used as one of the important markers for the detection of bladder cancer. The results of this study revealed that the serum VECF and BTA levels of patients after treatment were significantly lower than before treatment, and the serum marker levels of the study group were significantly lower than those of the control group. It is suggested that the TCM used in this study can effectively inhibit tumor progression and reduce tumor vitality. Long-term accumulation of toxins and glycation end products in patients with bladder cancer will increase the body's inflammatory factors, such as IL-6, CRP, TNF-*α*, and other acute reactive proteins. IL-6 can induce the synthesis of acute-phase protein and participate in the body's inflammatory response. TNF-*α* can easily provoke adhesion and infiltration of neutrophils and monocytes [33]. CRP is an inflammatory response marker protein, regulated by TNF-*α*, IL-6, and other mediators. The results of this study showed that the levels of inflammatory factors IL-6, CRP, and TNF-*α* in the patients after treatment were significantly lower than before treatment, and the levels of inflammatory factors in the study group were significantly lower than those in the control group, indicating that the use of traditional Chinese medicine in this study can significantly improve the inflammatory state to achieve the purpose of inhibiting tumor metastasis and recurrence. The results of this study showed that the clinical efficacy of the study group was significantly higher than that of the control group; after treatment, the levels of CD3+, CD4+, CD8+, and CD4+/CD8+ in the two groups were significantly improved compared with those before treatment, and the difference between the study group and the control group was statistically significant (*P* < 0.05). After treatment, the quality of life of the two groups was significantly improved compared to that of before treatment, and the difference between the two groups was statistically significant (*P* < 0.05). After treatment, the three-year cumulative survival rate of the study group was significantly higher than that of the control group. The recurrence rate in the study group was significantly lower than that in the control group. It is suggested that traditional Chinese medicine nursing combined with bladder perfusion with hydroxycamptothecin for patients with non-muscle-invasive bladder cancer can effectively reduce the risk of postoperative recurrence, improve the quality of life, improve immune system function, enhance body immunity, and improve long-term survival. However, there is still a gap for improvements in this study. Small sample size may lead to a large probability of error in data deviation, so we hope to increase the sample size in future research to reduce the deviation of results. Moreover, the causes for the decrease of adverse drug reactions shall be further explored to get better outcomes. These are the directions of our follow-up and improvement, so as to find a better remedy for this condition.

In summary, the clinical effects of Chinese medicine adjuvant treatment of bladder cancer patients after general anesthesia combined with epidural anesthesia and resection are significant. The levels of tumor markers and inflammatory factors are significantly reduced, the recurrence rate of patients after surgery is reduced, and the prognosis of patients is relatively ideal.

## Figures and Tables

**Table 1:** Comparison of clinical efficacy between the two groups of patients after treatment (*n* (%)).

Group	*n*	CR	PR	SD	PD	Total effective rate
Study group	80	25	42	9	4	67 (83.75)
Control group	80	11	36	21	12	47 (58.75)

**Table 2:** Comparison of the quality of life of the two groups of patients before and after treatment $\left(\bar{x}\pm s\right)$.

Index	Study group (*n* = 80)	Control group (*n* = 80)	*t*	*P*
*Physical function*				
Before treatment	54.32 ± 4.36	55.25 ± 4.47	1.223	>0.05
After treatment	75.63 ± 5.42	62.37 ± 5.11	6.742	<0.05

*Role function*				
Before treatment	51.25 ± 5.63	51.77 ± 5.71	0.651	>0.05
After treatment	78.46 ± 6.52	64.71 ± 6.27	7.384	<0.05

*Affective function*				
Before treatment	53.54 ± 5.46	54.08 ± 5.51	0.877	>0.05
After treatment	79.66 ± 6.85	66.28 ± 6.42	11.393	<0.05

*Cognitive function*				
Before treatment	56.34 ± 5.47	55.89 ± 5.26	1.027	>0.05
After treatment	72.34 ± 6.67	63.71 ± 6.27	5.314	<0.05

*Social function*				
Before treatment	51.43 ± 4.15	51.22 ± 4.23	0.243	>0.05
After treatment	78.96 ± 6.53	63.38 ± 5.22	12.731	<0.05

**Table 3:** Comparison of the recurrence rate between the two groups of patients during the three-year follow-up (*n* (%)).

Group	*n*	Follow-up for 1 year	Follow-up for 2 years	Follow-up for 3 years
Study group	80	7 (8.75)	17 (21.25)	26 (32.50)
Control group	80	13 (16.25)	29 (36.25)	49 (61.25)

## Data Availability

The datasets during the current study are available from the corresponding author upon reasonable request.
